# CD44 cross-linking increases malignancy of breast cancer via upregulation of p-Moesin

**DOI:** 10.1186/s12935-020-01663-4

**Published:** 2020-11-23

**Authors:** Song Hu, Xiaoxing Shi, Yiwen Liu, Yiqing He, Yan Du, Guoliang Zhang, Cuixia Yang, Feng Gao

**Affiliations:** 1 Department of Molecular Biology, Shanghai Jiao Tong University Affiliated Sixth People’s Hospital, Shanghai, 200233 China; 2 Department of Laboratory Medicine, Shanghai Wujing General Hospital, Shanghai, 201103 China; 3 Department of Clinical Laboratory, Shanghai Jiao Tong University Affiliated Sixth People’s Hospital, Shanghai, 200233 China

**Keywords:** Breast cancer, CD44 cross-linking, p-Moesin, Migration, Invasion

## Abstract

**Background:**

CD44 is highly expressed in most cancer cells and its cross-linking pattern is closely related to tumor migration and invasion. However, the underlying molecular mechanism regarding CD44 cross-linking during cancer cell metastasis is poorly understood. Therefore, the purpose of this study was to explore whether disruption of CD44 cross-linking in breast cancer cells could prevent the cells migration and invasion and determine the effects of CD44 cross-linking on the malignancy of the cancer cells.

**Methods:**

The expression of CD44, CD44 cross-linking and Moesin phosphorylation in breast cancer cells was assessed by Western Blot assays. Effects of CD44 cross-linking on tumor metastasis were evaluated by Transwell assay. The effects of CD44 cross-linking disruption on cell viability were assessed using CCK-8 assays. The expression of p-Moesin between normal and breast cancer tissues was examined by immunohistochemical staining.

**Results:**

High expression of CD44 cross-linking was found in invasive breast cancer cells (BT-549 and MDA-MB-231), which is associated with the malignancy of breast cancer. The expressions of ERM complex in a panel of breast cancer cell lines indicate that Moesin and its phosphorylation may play a significant role in cell metastasis. Moesin phosphorylation was inhibited by CD44 de-crosslinking in breast cancer cells and Moesin shRNA knockdown attenuated the promotion of CD44 cross-linking on cell migration and invasion. Finally, immunohistochemistry results demonstrated that p-Moesin was overexpressed in primary and metastatic cancers.

**Conclusions:**

Our study suggested that CD44 cross-linking could elevate p-Moesin expression and further affect migration and invasion of breast cancer cells. These results also indicate that p-Moesin may be useful in future targeted cancer therapy.

## Background

Malignant tumor metastasis is usually the leading cause of death in cancer patients [1]. Breast cancer is a multistate tumor that often has fatal effects by metastasizing to distant organs, such as bones, lungs, and liver [2–4]. In light of this, it is critical to understand the regulatory mechanisms driving breast cancers (BrCas) metastasis and discover new therapeutic targets.

As one of the adhesion molecules related to tumor metastasis, CD44, a receptor of hyaluronan (HA), is becoming attractive for its role as a stem cell marker [5, 6]. Until now, the function of CD44 in cancers is not fully understood, although data have proved that CD44 activities are usually triggered by binding with its ligands, especially HA. In fact, the interaction of receptors with ligands has always been a focus regarding the cellular behaviors under pathological processes, like inflammation, immune responses, wound healing and cancer progression [7–9]. As HA is a non-sulfated glycosaminoglycan which usually binds with the multiple sites on the receptors that can cause CD44 linking together, we and other studies have found that CD44 cross-linking and de-crosslinking can deliver opposite signals to cells. Unfortunately, little is known about CD44 self-linking in breast malignancy.

CD44 cross-linking was initially described in rat pancreatic cancer cells, which can improve the ligand HA binding capacity [10]. Subsequent researches showed that CD44 cross-linking has been associated with the progression of other human cancers. For example, in neuroblastoma cells, clusters of CD44 are located at the filamentous pseudopods and focal globular, which facilitate migration and invasion into the brain [11]. CD44 clustering can also promote BrCas metastasis by relocating metalloproteinase-9 and up-regulating LFA-1 and VLA-4 [12, 13]. However, most of the studies were conducted in a CD44 antibody-mediated cross-linking manner, so a model that naturally mimics the in vivo clustering of CD44 is urgently needed for cancer study. Our previous studies showed that high molecular weight HA can stimulate CD44 cross-linking in BrCas cells [14] and the HA contents are more abundant in invasive BrCas cells [15]. These prompted us to further investigate the role of HA dependent CD44 self-linking in BrCas progression and its underlying mechanisms.

In addition to CD44, the ERM proteins (Ezrin/Radixin/Moesin) [16], which act as cross-linkers between the actin cytoskeleton and intracellular domain of CD44, have been implied in cell adhesion and motility [17]. Among them, Moesin is particularly attractive for its curial role in organizing membrane domains and receptor signaling, as well as regulating the metastasis of tumor cells. Evidence has proved that CD44-Moesin interaction promotes human brain tumor proliferation by activating the Wnt signaling pathway [18]. Other reports suggested that the expression of Moesin in glioma cells was significantly higher than that in normal astrocytes [19] and there was a strong negative correlation with progression-free survival and overall survival [20]. However, no role for Moesin activation in BrCas progression has been defined. Therefore, it is reasonable to speculate that CD44 receptors self-linking or aggregations are closely associated with the intracellular ERM molecules, and it is necessary to explore the regulating mechanism of ERM on CD44 interactions.

In this study,we aimed to determine the role of CD44 cross-linking on BrCas metastasis and explore the underlying mechanisms. We first analyzed the expression of CD44 cross-linking in BrCas cell lines, and elucidated the relations between CD44 clustering status and BrCas metastasis. Then, we investigated the effect of CD44 cross-linking on downstream ERM proteins. Our data showed that CD44 clustering could promote BrCas malignancy and disruption of CD44 clustering could dramatically inhibit cancer cell migration and invasion. Notably, phosphorylated Moesin (p-Moesin) may play an important role in CD44 cross-linking. These findings not only provide new mechanistic insights into CD44 cross-linking in BrCas but also indicate that p-Moesin may be a potential therapeutic target for treating invasive BrCas.

## Materials and methods

### Cell culture and antibodies

Human BrCas cell lines (MDA-MB-453, MCF-7, T-47D, MDA-MB-231, BT-549 and Hs-578t) were purchased from the Cell Bank of the Type Culture Collection of the Chinese Academy of Sciences (Shanghai, China). MDA-MB-453 cells were cultured in L-15(Gibco, USA), MCF-7 cells were cultured in MEM(Gibco, USA); BT-549 cells were cultured in RPMI-1640 medium(Gibco, USA); and MDA-MB-231, T-47D and Hs-578t cells were cultured in high-glucose DMEM (Gibco, USA).All the media were supplemented with 10% fetal bovine serum(Gibco, USA) and 100 IU/ml penicillin/streptomycin. Additional insulin with a final concentration of 0.01 mg/ml was added to the culture medium of BT-549 and Hs-578t. All cell lines were cultured at 37 °C in humidified air with 5% CO_2_ and 95% air. Primary antibodies against CD44 (Abcam, ab119348), CD44 (clone IM7, Cat #14-0441-86), ERM (CST, 3142S), p-ERM (CST, 3141S), Moesin (Abcam, ab52490) p-Moesin (Abcam, ab177943), p-Merlin(CST, 13281S), Merlin (CST, 12888) were used.

### Quantitative real-time PCR (qRT-PCR)

Total RNA was extracted from cultured cells (MCF-7, T-47D, BT-549, and MDA-MB-231) by RNAiso Plus (Takara, Japan). RNA (1 μg) was reverse transcribed with the PrimeScript™ RT Reagent Kit with gDNA Eraser (Takara, Japan). Real-time PCR assays were performed by using SYBR Green Mix (Takara, Japan) according to the manufacturer’s protocol. All qRT-PCR values of each gene were normalized against that of GAPDH. The relative expression of genes was calculated by the 2^−ΔΔCt^ method.

The primer sequences for qRT-PCR are: CD44-F: GACACCATGGACAAGTTTTGG, CD44-R: GACACCATGGACAAGTTTTGG; Moesin-F, ATGCCCAAAACGATCA GTGTG, Moesin-R: ACTTGGCACGGAACTTAAAGAG; Ezrin-F: ACCAATCAA TGTCCGAGTTACC, Ezrin-R: GCCGATAGTCTTTACCACCTGA; Radixin-F: AA TTGTGGCTAGGTGTTGATGC, Radixin-R: GGTGCCTTTTTGTCGATTGGC; Merlin-F: AGTGGCCTGGCTCAAAATGG, Merlin-R: TGTTGTGTGATCTCCTGA ACCA; GAPDH-F: AGCCTCAAGATCATCAGC, GAPDH-R: GAGTCCTTCCAC GATACC.

### RNA interference

The shRNA-carrying lentiviruses against Moesin, and negative control were produced by Genechem (Shanghai, China). BT-549 and MDA-MB-231 cells were infected with concentrated virus according to the manufacturer’s protocol, and the expression of Moesin was validated by western blot analysis.

### Western blotting

RIPA buffer (Beyotime, China) was used for protein extraction. After the total protein concentration was determined by a bicinchoninic acid protein assay kit (Sigma, USA), 30 μg protein samples were separated by 8% SDS polyacrylamide gels and transferred onto PVDF membranes(Millipore, Billerica, USA). The membrane was blocked with 5% nonfat milk in TBST for 1 h and incubated with the indicated antibody at 4 °C overnight. Then HRP-conjugated secondary antibodies (1:5000) were added. Bands were subsequently visualized using the enhanced plus chemiluminescence assay (Pierce, USA). Measurement of the bands was conducted on an ImageQuant LAS 4000 mini.

### Cell proliferation

In brief, cells were treated with 300 μg/ml hyaluronidase and control medium. Then, equal numbers of cells (2000 cells/well) were seeded into 96-well plates for the proliferation experiment. The proliferation of MDA-MB-231 and BT-549 cells was measured via CCK-8 assay (KeyGen Biotech,China) according to the manufacturer’s protocol.

### CD44 Cross-linking

Cells were treated with different concentrations of hyaluronidase for 0.5 h, then the cells were washed 3 times with ice-cold PBS (20 mM sodium phosphate, 0.15 M NaCl, pH 8.0). CD44 cross-linking was performed by incubation with 2 mM bis (sulfosuccinimidyl) suberate (BS_3_) (Pierce, USA) for 30 min and quenched by incubation with 20 mM Tris, pH 7.6 for 15 min at room temperature. Cells were washed twice with PBS and lysed with cell lysis buffer.

For antibody-mediated CD44-crosslinking. BrCas cells were incubated for 90 min at 37 °C with rat monoclonal CD44 antibody (10 mg/ml) (clone IM7, Cat #14-0441-86). After three washes, cells were incubated with 1 mg/ml of goat anti-rat IgG-Fc (Cat #31226) for 60 min at 37 °C and then subjected to cell lysis.

### Cell migration and invasion assays

To evaluate the migration and invasion abilities of BrCas cells, Transwell assays were performed. In brief, MCF-7, T-47D, MDA-MB-231 and BT-549 cells were suspended in medium containing 5% FBS after transfection. In addition, 3 × 10^4^ cells were seeded into the upper chamber of an 8-μm pore size insert with or without Matrigel (BD Biosciences, USA). The chambers were deposited in a 24-well plate with 600 μl of 20% FBS medium. After 24 h’s incubation, the cells were fixed with 4% paraformaldehyde (Beyotime, China) and stained with crystal violet (Beyotime, China). After removing the cells on the upper surface of the chamber, the penetrated cells were captured and the number of cells was counted by Image J software at 200× magnification in five random fields under a microscope.

### Immunohistochemistry (IHC) and staining evaluation

Formalin-fixed paraffin-embedded human BrCas tissues were obtained from Shanghai Superchip Biotech (HBreD055CD01). The expression of p-Moesin in the tissue chip of the cohort of 55 tissues was examined by IHC. The tissue chip was incubated with rabbit monoclonal antibody against human p-Moesin (Abcam, ab177943). Then the sections of tissue chip were detected by Outdo Biotech (Shanghai, China).

The expression of Moesin was scored with intensity of staining and the percentage of the cells of interest staining. We divided the intensity of staining into 4 groups: 0 (–), 1 (+), 2 (++), and 3(+++). The (–), (+), (++), (+++) were defined as no staining, weak staining, moderate staining and intense staining. Then we ranked the percentage of positive staining into 4 categories: 0 (0%), 1 (1–29%), 2 (30–69%), and 3 (≥ 70%) as shown in Additional file 1: Figure S4. The IHC scores are obtained by multiplying the above two scores. The final figures were created by Graphpad 7.

### Statistical analysis

All data are presented as the mean ± SD and were analyzed with GraphPad Prism 7 and SPSS v23 software. The student’s t-test was used to identify the differences between the treated groups and their controls. IHC scores among three groups were analyzed with the non-parametric alternative to ANOVA. A P value < 0.05 was considered statistically significant in the text and figures (*P < 0.05, **P < 0.01, ***P < 0.001).

## Results

### CD44 cross-linking in breast cancer

**Fig. 1:**
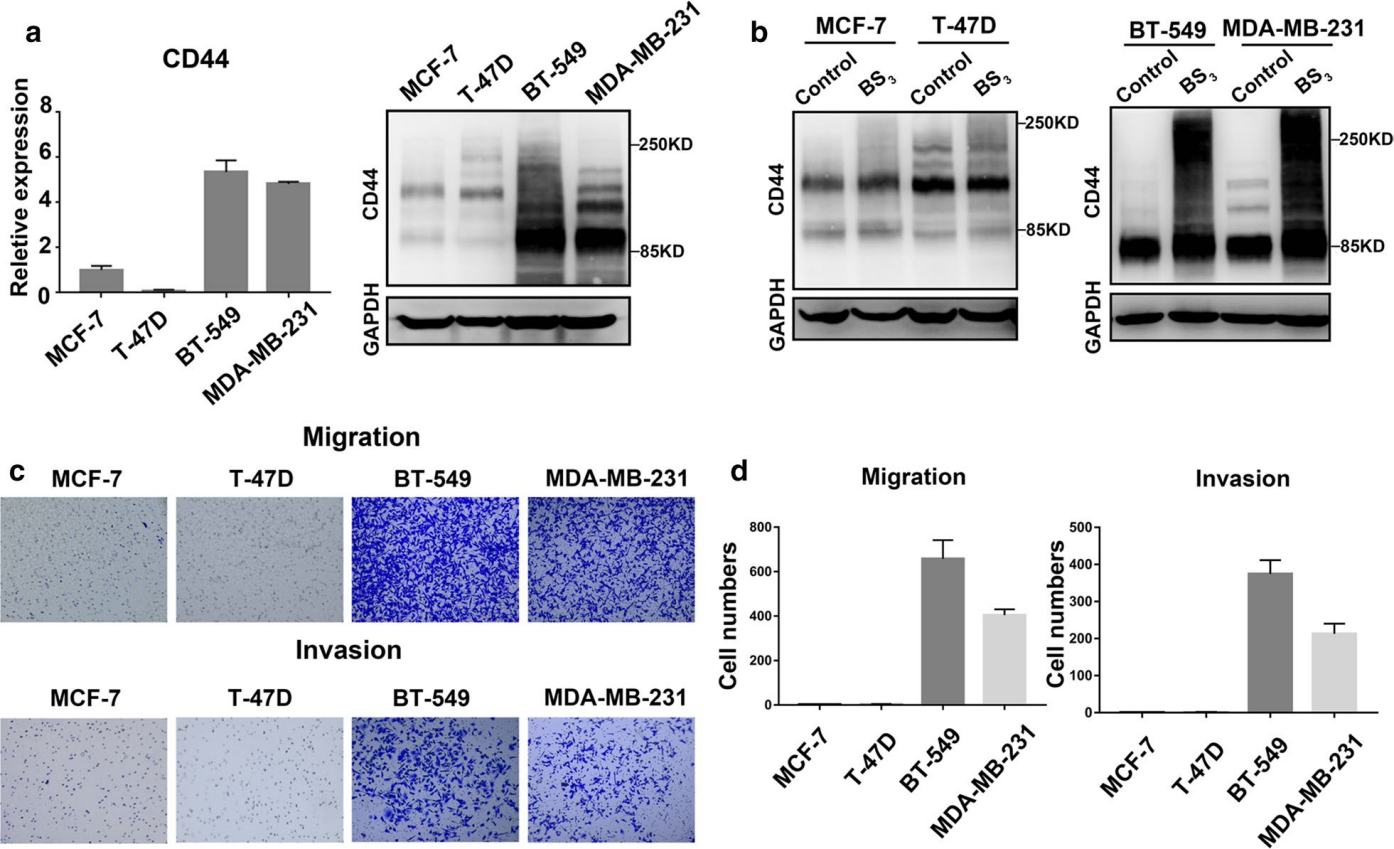
CD44 cross-linking in breast cancer. **a** Expression of CD44 in breast cancer cell lines MCF-7, T-47D, BT-549 and MDA-MB-231. On the left, qRT-PCR results of CD44,on the right, western blot analysis of CD44; **b** Expression of CD44 cross-linking in breast cancer cell lines; **c**, **d** Comparison of migration and invasion in breast cancer cell lines

Since there are few studies involving CD44 cross-linking abnormalities in BrCas, we selected four BrCas cell lines with different degrees of malignancy to explore the expression of CD44 and CD44 cross-linking. We found that the expression of CD44 was relatively higher in invasive BrCas cells MDA-MB-231 and BT-549, and lower in MCF-7 and T-47D cells (Fig. 1a). In addition, to show the CD44 cross-linked status on BrCas cells, we treated the cells with BS_3_, a membrane-impermeable compound that cross-link only nearby proteins located within the length of the spacer. After incubation with BS_3_ or control buffer, obvious cross-linked CD44 aggregations were showed in MDA-MB-231 and BT-549 cells rather than in MCF-7 and T-47D cells (Fig. 1b). At the same time, Transwell experimental results showed that MDA-MB-231 and BT-549 presented higher abilities of migration and invasion than MCF-7 and T-47D (Fig. 1c and d). These data suggest that CD44 expression level and CD44 cross-linking abilities may be accompanied by BrCas metastasis.

### CD44 cross-linking is associated with the malignancy of breast cancer

**Fig. 2:**
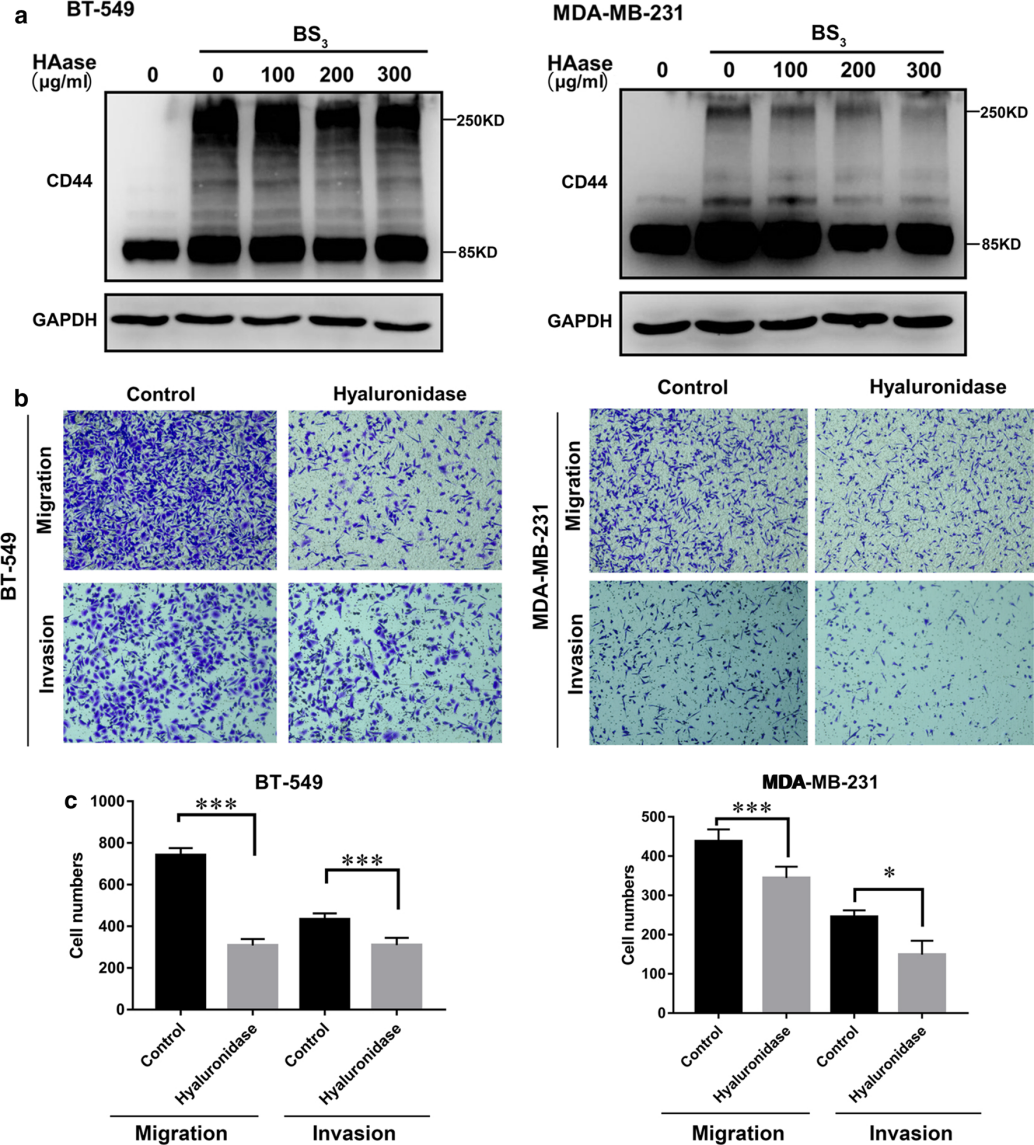
CD44 cross-linking is associated with the malignancy of breast cancer. **a** The effects of different concentrations of hyaluronidase on CD44 cross-linking of BT-549 and MDA-MB-231 cells were detected by Western Blot assay. **b**, **c** The effect of 300 μg/ml hyaluronidase on the cell migration and invasion ability of BT-549 and MDA-MB-231 cells; *P < 0.05, ***P < 0.001

To investigate the effect of CD44 cross-linking on BrCas cells, we first carried out experiments to disconnect the cross-linking using three reagents (oligosaccharides of hyaluronan, hyaluronidase and Latrunculin B). All of them have been proven to effectively block the CD44 cross-linking [14, 21]. The results showed that hyaluronidase has the best dis-connecting effect and is not toxic to cells (Additional file 2: Figure S1), in which 300 μg/ml was selected as the effective concentration in our experiment. Next, we treated BrCas cells with hyaluronidase to abrogate CD44 cross-linking (Fig. 2a). Our studies showed that the removal of CD44 cross-linking in BT-549 and MDA-MB-231 cells significantly reduced cell migration and invasion without changing CD44 expression (Fig. 2b and c), suggesting that CD44 cross-linking may play an important role in the malignancy of BrCas.

### Moesin phosphorylation was inhibited by CD44 de-crosslinking in breast cancer cells

**Fig. 3:**
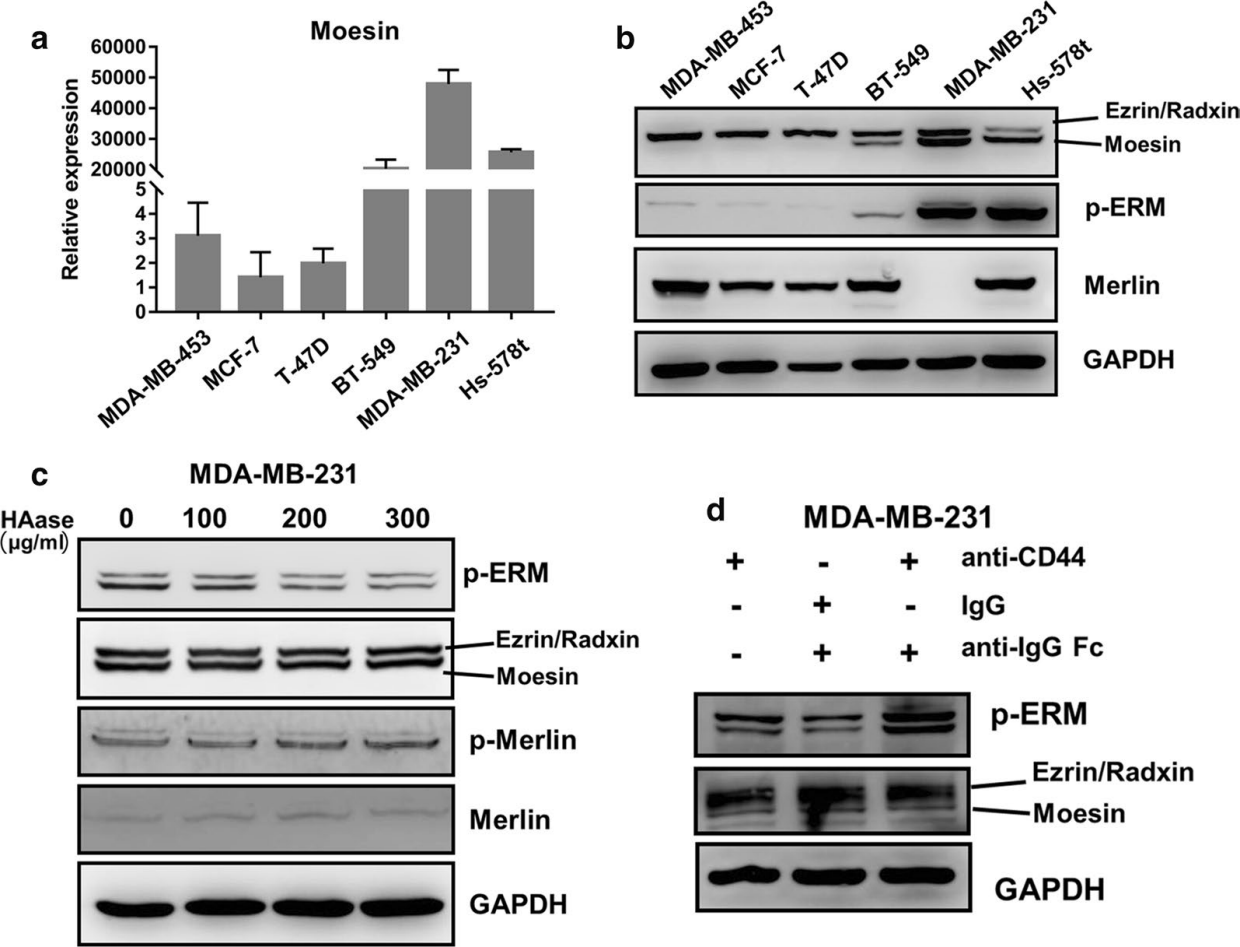
Moesin phosphorylation can be affected by CD44 cross-linking in breast cancer cells. **a** Detection of Moesin expression in breast cancer cell lines by qRT-PCR; **b** Detection of ERM, p-ERM and Merlin expression in breast cancer cell lines by western blot; **c** Effects of hyaluronidase at different concentrations on the expression of ERM and Merlin and their phosphorylated forms; **d** p-Moesin and ERM expression after CD44 antibody mediated cross-linking in MDA-MB-231 cells

Since CD44 cross-linking is an abnormal result of receptor-ligand interaction, and most of cellular activities are conducted through downstream ERM complex, we further explore the changes of intracellular cytoskeletal molecules underneath the CD44 receptor. Firstly, we screened the expressions of ERM complex in a panel of BrCas cell lines. qRT-PCR and Immunoblot analysis both showed that the expression of Moesin was relatively higher in invasive BrCas cells MDA-MB-231, Hs-578t and BT-549, and lower in MDA-MB-453, MCF-7 and T-47D cells (Fig. 3a and b). Interestingly, p-Moesin was overexpressed in invasive breast cancer cells (Fig. 3b). However, there was no significant difference in the expression of the other three actin-membrane crosslinkers (Ezrin, Radixin and Merlin) (Fig. 3b and Additional file 3: Figure S2). These results indicated that Moesin and its phosphorylation may play a significant role in cell metastasis. However, to our surprise, when we destroyed the CD44 cross-linking in MDA-MB-231 and BT-549, we found that it was the phosphorylation of Moesin, rather than Moesin expression, that was inhibited (Fig. 3c and Additional file 4: Figure S3A), suggesting that CD44 cross-linking may induce Moesin activation only, not the expression. To further clarify this hypothesis, we used antibody to re-cross link CD44. The addition of the secondary antibody enhanced CD44 cross-linking and resulted in increased p-Moesin expression (Fig. 3d and Additional file 4: Figure S3B), which suggested that CD44 cross-linking could promote p-Moesin expression. Also we can see that Ezrin, Radxin and Merlin and their phosphorylated forms do not show significant differences (Fig. 3c and Additional file 4: Figure S3A). Taken together, these results suggested that CD44 cross-linking may regulate tumor cell migration and invasion by altering the phosphorylation of Moesin.

### Moesin shRNA knockdown attenuated the promotion of CD44 cross-linking on cell metastasis and invasion

**Fig. 4:**
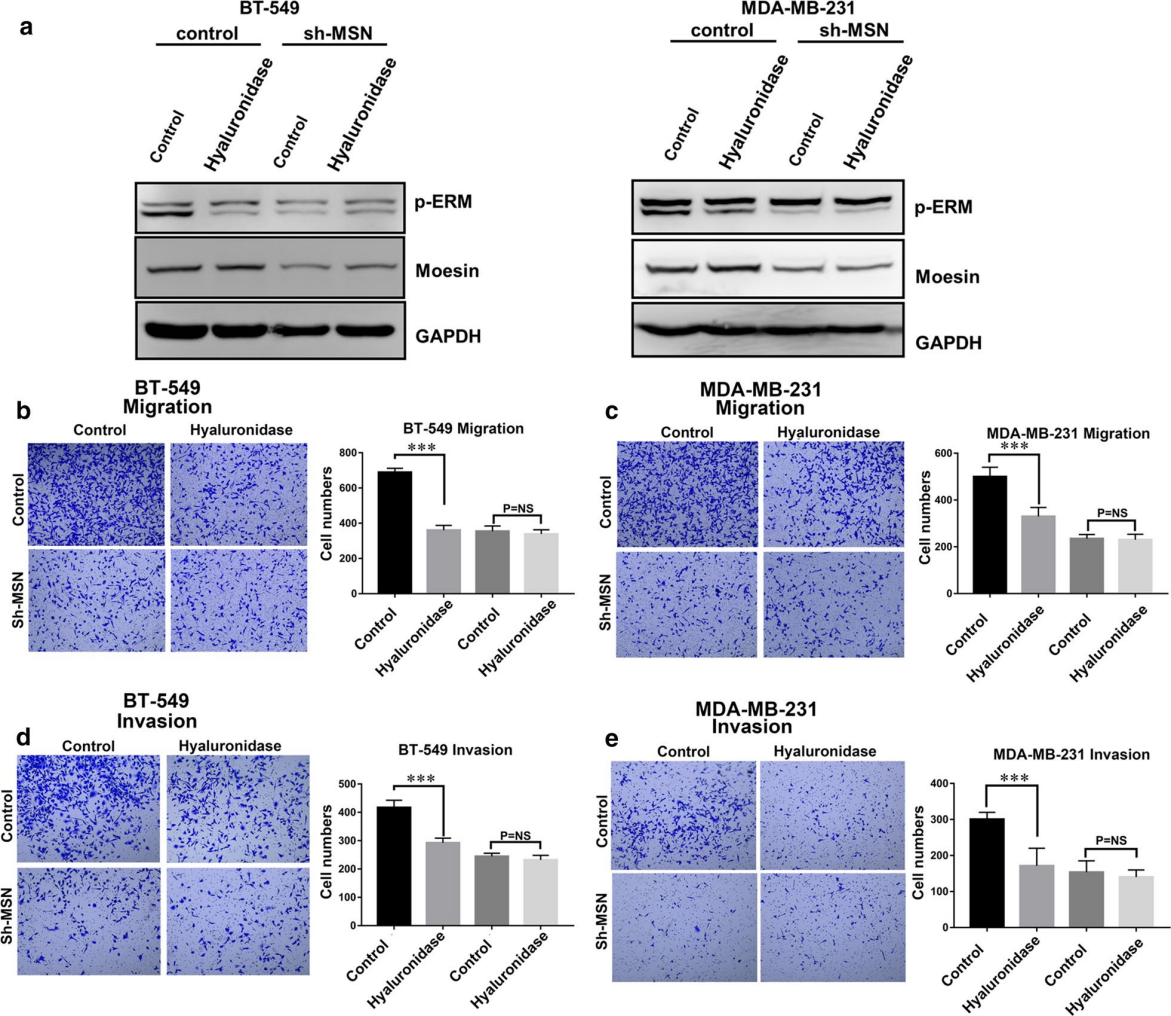
Moesin shRNA knockdown attenuated the promotion of CD44 cross-linking on cell metastasis and invasion. **a** p-Moesin and Moesin expression of BT-549 and MDA-MB-231 and Moesin knockdown cells after treatment with hyaluronidase. **b**, **c** Migration of BT-549 and MDA-MB-231 and Moesin knockdown cells after treatment with hyaluronidase; **d**, **e** Invasion of BT-549 and MDA-MB-231 and Moesin knockdown cells after treatment with hyaluronidase; ***P < 0.001, *NS* no significance

To further clarify that CD44 cross-linking indeed regulates tumor cell migration and invasion by affecting p-Moesin in BrCas, we reduced Moesin expression in MDA-MB-231 and BT-549 by RNA interference. Western blot analysis showed that the down-regulation of Moesin could simultaneously reduce the expression of p-Moesin (Fig. 4a). Through Transwell experiment, we analyzed the effect of p-Moesin down-regulation on cell migration and invasion. The results showed that after p-Moesin was down-regulated in BrCas cells, cell migration and invasion were inhibited despite the presence of CD44 cross-linking (Fig. 4b and c), and the same results were observed in cell invasion experiments (Fig. 4d and e). These results suggest that p-Moesin expression plays an important role in affecting the migration and invasion of BrCas cells after CD44 cross-linking.

### p-Moesin was overexpressed in primary and metastatic cancers

**Fig. 5:**
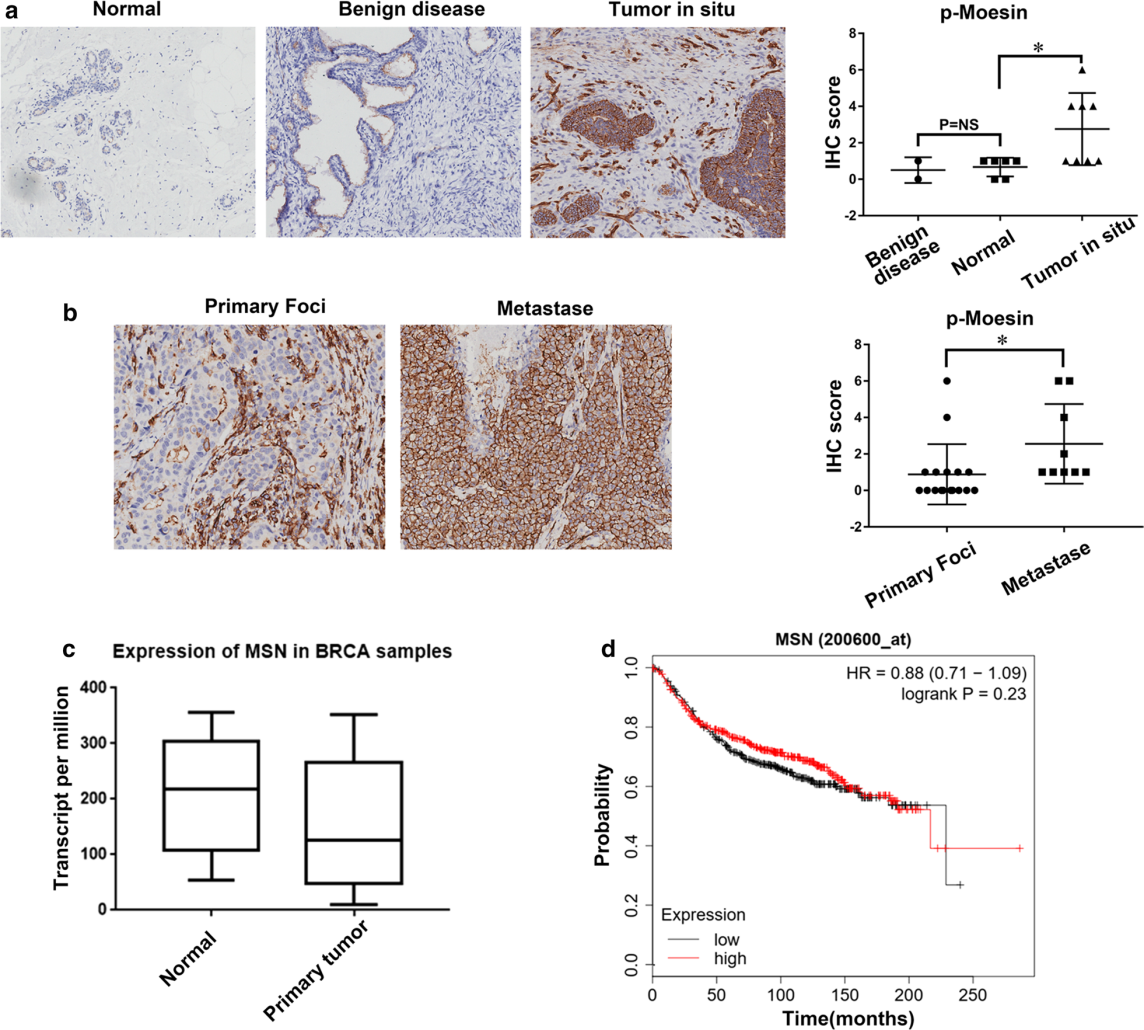
p-Moesin was overexpressed in primary and metastatic cancers. **a**, **b** Representative images from a microarray of human breast tissue that includes tumor and adjacent normal tissues stained by IHC for p-Moesin, IHC scores of p-Moesin were on the right; **c** Expression levels of Moesin in cancer and adjacent normal tissues of breast cancer in TCGA database; **d** Moesin expression has no significant correlation with patient overall survival in TCGA database,*P < 0.05

As our data show that p-Moesin may play a role in BrCas metastasis, we next try to investigate whether p-Moesin could be used in the clinicopathologic detection in BrCas. We analyzed the p-Moesin expression levels in 55 samples, including 34 BrCas (8 ductal breast carcinoma in situ and 26 invasive ductal breast carcinoma), 2 benign breast disease, 13 lymph nodes and 6 adjacent normal tissues by IHC staining assays. The data showed that p-Moesin level was significantly higher in ductal breast carcinoma in situ than those in adjacent normal tissues (Fig. 5a). Accordingly, the expression of p-Moesin in metastase tissues was also higher than that in primary foci of invasive ductal breast carcinoma (Fig. 5b). To further identify the significances between p-Moesin and Moesin, we carried on a TCGA database method to analyze the expression levels of Moesin in BrCass. We found that there were no significant differences in the expression of Moesin between cancer and adjacent tissues (Fig. 5c). Similarly, there was no difference in survival between patients with different Moesin expression (Fig. 5d). These data suggested that the phosphorylated form of Moesin not Moesin is correlated with tumor progression and metastasis and may be applied in the future clinicopathologic detection (Additional file 5: Figure S5).

## Discussion

Receptor cross-linking is often considered to be a hallmark of malignant tumor initiation and progression, during which downstream signal pathways are usually activated that affect cell function [22–24]. CD44, the main receptor for HA, is a well-accepted molecular marker for cancer stem cells. Data have stated that CD44 is involved in tumor-initiating that includes CD44 self-interaction and downstream signal activation [25]. However, the mechanism of CD44 clustering in cancer progression is not well illustrated. In this study, we demonstrate for the first time that CD44 cross-linking promotes BrCas cell aggression by regulating the downstream ERM proteins, particularly through p-Moesin. Correspondingly, the decrease of p-Moesin could inhibit the BrCas cells migration and invasion after disrupting CD44 cross-linking, suggesting a CD44 clustering-p-Moesin pathway that regulates BrCas malignancy (Additional file 5: Figure S5).

Tumor cell metastasis is one of the features of most lethal tumors [26]. Studies have found that CD44 is overexpressed in metastatic BrCas [27] and its multiple spliced forms have also been shown to be closely associated with BrCas cell metastasis [28]. Besides overproduction, CD44 cross-linking is also believed to induce cancer cell adhesion and migration [13]. However, a recent study stated that CD44 clustering in breast epithelial cells could reduce cell growth [29]. These studies suggest that the functions of CD44 cross-linking are diverse and unknown details are needed to be explored.

As reported, CD44 molecules are dispersed over the cell surface under homeostatic conditions, whereas clustering could be induced by aberrant HA deposition in pathological microenvironments [14, 29]. Intriguingly, such receptors self-linking could be reversed following the degradation process by abnormal metabolites, such as hyaluronidase. The significances of CD44 receptors self-aggregation and de-cross- linking are being investigated. Some observations have suggested that the disruption of CD44 self-linking could inhibit BrCas cells migration and invasion [30, 31]. However, the mechanisms involved are not elucidated thoroughly, including the downstream molecules closely connected to CD44 or intracellular skeletal complex beneath the CD44 receptors.

It is well known that ERM complex (Ezrin, Radixin and Moesin) are cytoskeletal molecules connected to CD44 C-terminal domain and are believed to be closely related to cancer malignancy. Due to their structure homology, the three members are often studied as a whole [32]. However, a growing body of evidence proves that ERM proteins perform distinct functions during different biological processes [19, 33–35]. In our study, we found that CD44 cross-linking could cause Moesin phosphorylation without changing its expression. At the same time, Ezrin and Radixin, two other components of the ERM complex showed no alterations in quantity and activity, suggesting that CD44 cross-linking plays its downstream role mainly through p-Moesin in BrCas. Previous findings indicated that HA can significantly enhance the invasiveness of glioma cells by promoting the binding of CD44 and Moesin [36]. Nevertheless, in our current study, we found that HA increases BrCas malignancy through inducing CD44 receptors self-linking, thus triggering downstream Moesin’s phosphorylation, rather than simply binding to CD44. Moreover, our loss-and-gain-of-function experiment proved that removing ligand (HA) with hyaluronidase to destroy CD44 cross-linking obviously inhibited p-Moesin expression, while p-Moesin was rescued following the addition of an antibody that re-induced CD44 cross-linking. Since CD44 clustering does not affect the expression of Moesin, we knocked down Moesin to downregulate p-Moesin. We observed that the promoting effect of CD44 clustering on BrCas cell migration could be halted after the Moesin knockdown. Taken together, these data suggested that p-Moesin may be indispensable in BrCas cell aggression caused by CD44 cross-linking and may have potential clinical application value.

To further verify the clinical significance of p-Moesin in BrCas, we determined its expression level with a sample cohort. Our IHC results showed that p-Moesin is highly expressed in primary BrCas compared with normal tissue. Additionally, we also observed that the p-Moesin expression in metastases is higher than that in primary tumors, suggesting that p-Moesin is clinically associated with the induction of metastasis in BrCas. Furthermore, through analyzing the TCGA database, we found that there was no significant difference in Moesin expression between BrCas and adjacent tissues, as well as no correlation of Moesin with BrCas patients’ poor prognosis. Collectively, our study highlighted that p-Moesin rather than Moesin, could be applied as a potential target for BrCas therapy.

## Conclusions

Our study demonstrated a novel finding of CD44 cross-linking with BrCas cells malignancy. The downstream mechanism revealed that a CD44 associated cytoskeletal protein, Moesin, was responsible for CD44 aggregation during BrCas metastasis when phosphorylation occurred. Further, we suggested that p-Moesin but not Moesin is overexpressed in primary BrCas and may be useful in future targeted cancer therapy.

## Supplementary information

**Additional file 1: Figure S4.** Representative images and quantification of p-Moesin staining. The intensity of IHC staining were ranked into 4 grades: 0 (–), 1 (+), 2 (++), and 3 (+++) as indicated.

**Additional file 2: Figure S1.** Effect of hyaluronidase on CD44 crosslinking and cell proliferation. (A) Effects of oHA, hyaluronidase and Lat B on CD44 cross-linking; (B) BT-549 and MDA-MB-231 were treated with 300 μg/ml hyaluronidase, and their proliferation capacity was detected by CCK-8 assay.

**Additional file 3: Figure S2.** Expression of Ezrin, Radixin and Merlin in breast cancer cell lines. (A-C) Expression levels of Ezrin, Radixin and Merlin in six breast cancer cell lines.

**Additional file 4: Figure S3.** Alteration of CD44 cross-linking status on the expression level of P-Moesin in BT-549 cells. (A) Effects of hyaluronidase at different concentrations on the expression of ERM and Merlin and their phosphorylated forms in BT-549 cells; (B) p-Moesin and ERM expression after CD44 antibody mediated cross-linking in BT-549 cells.

**Additional file 5: Figure S5.** Working model of the CD44 cross-linking : p-Moesin regulatory axis in breast cancer.

## Data Availability

The datasets used and/or analysed during the current study are available from the corresponding author on reasonable request.

## Abbreviations

HA: Hyaluronic acid
DMEM: Dulbecco’s modified Eagle medium
PBS: Phosphate buffered solution
TCGA: The cancer genome atlas
qRT-PCR: Quantitative real time polymerase chain reaction
IHC: Immunohistochemistry
PVDF: Polyvinylidene fluoride
CCK-8: Cell counting kit-8
shRNA: Short hairpin RNA
GAPDH: Glyceraldehyde-3-phosphate dehydrogenase
ERM: Ezrin/Radixin/Moesin
