# Supplementary figures and images for: CD44 cross-linking increases malignancy of breast cancer via upregulation of p-Moesin
Source: Cancer Cell Int. 2020 Nov 23;20:563. doi: 10.1186/s12935-020-01663-4 (PMC7686781; doi:10.1186/s12935-020-01663-4)

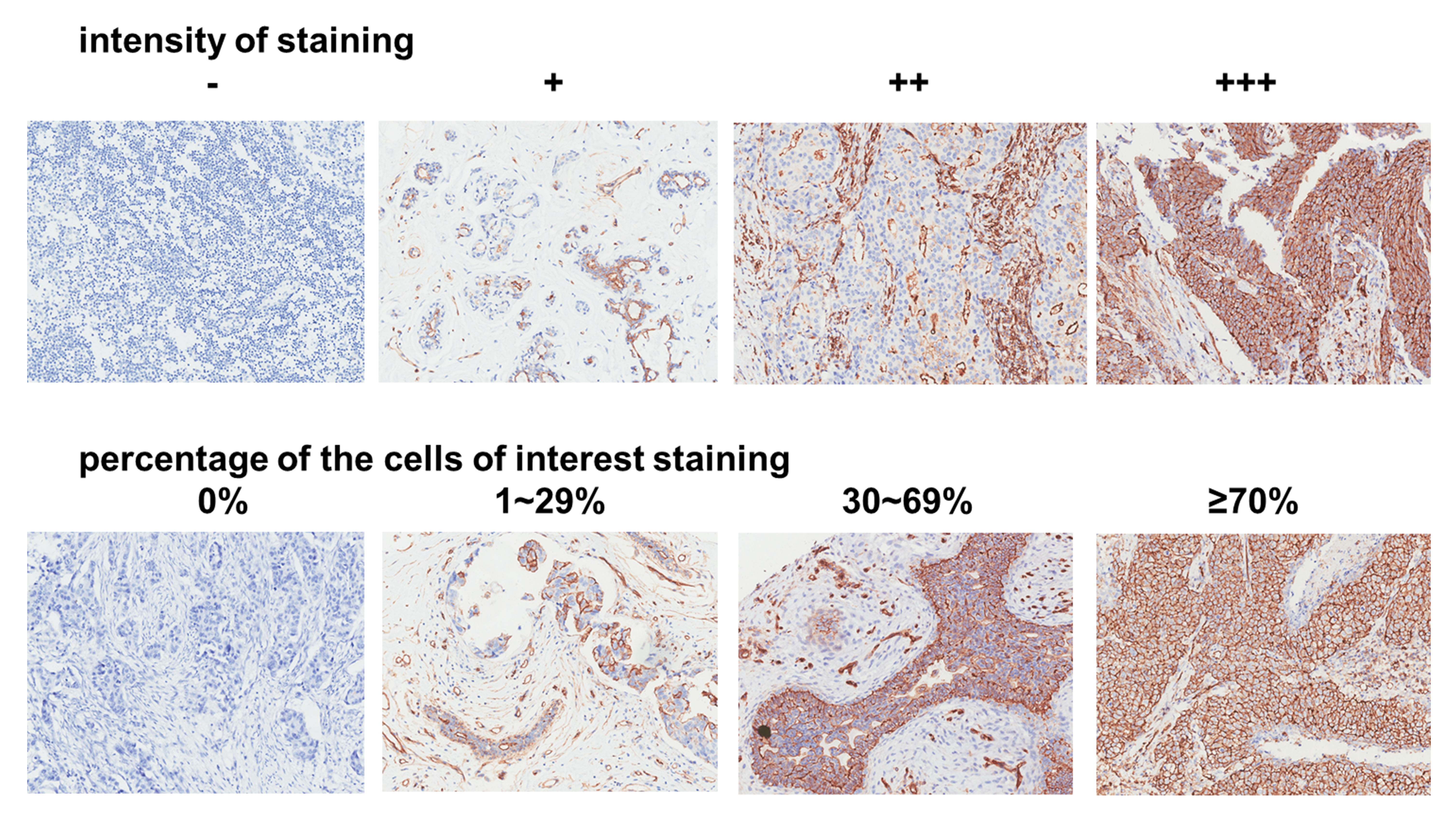

Supplement: Supplementary file 1 — Additional file 1: Figure S4. Representative images and quantification of p-Moesin staining. The intensity of IHC staining were ranked into 4 grades: 0 (–), 1 (+), 2 (++), and 3 (+++) as indicated. [file 12935_2020_1663_MOESM1_ESM.tif]

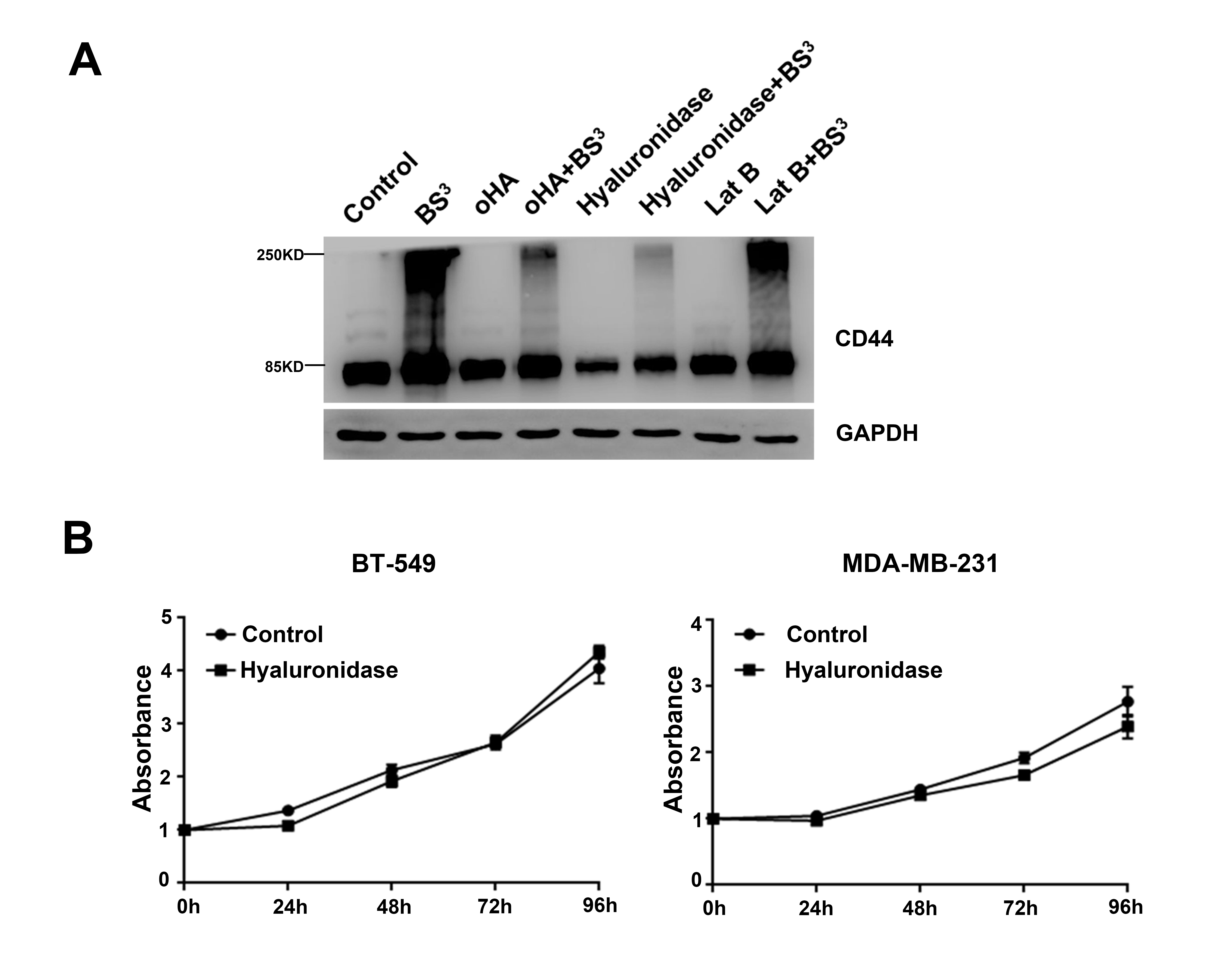

Supplement: Supplementary file 2 — Additional file 2: Figure S1. Effect of hyaluronidase on CD44 crosslinking and cell proliferation. (A) Effects of oHA, hyaluronidase and Lat B on CD44 cross-linking; (B) BT-549 and MDA-MB-231 were treated with 300 μg/ml hyaluronidase, and their proliferation capacity was detected by CCK-8 assay. [file 12935_2020_1663_MOESM2_ESM.tif]

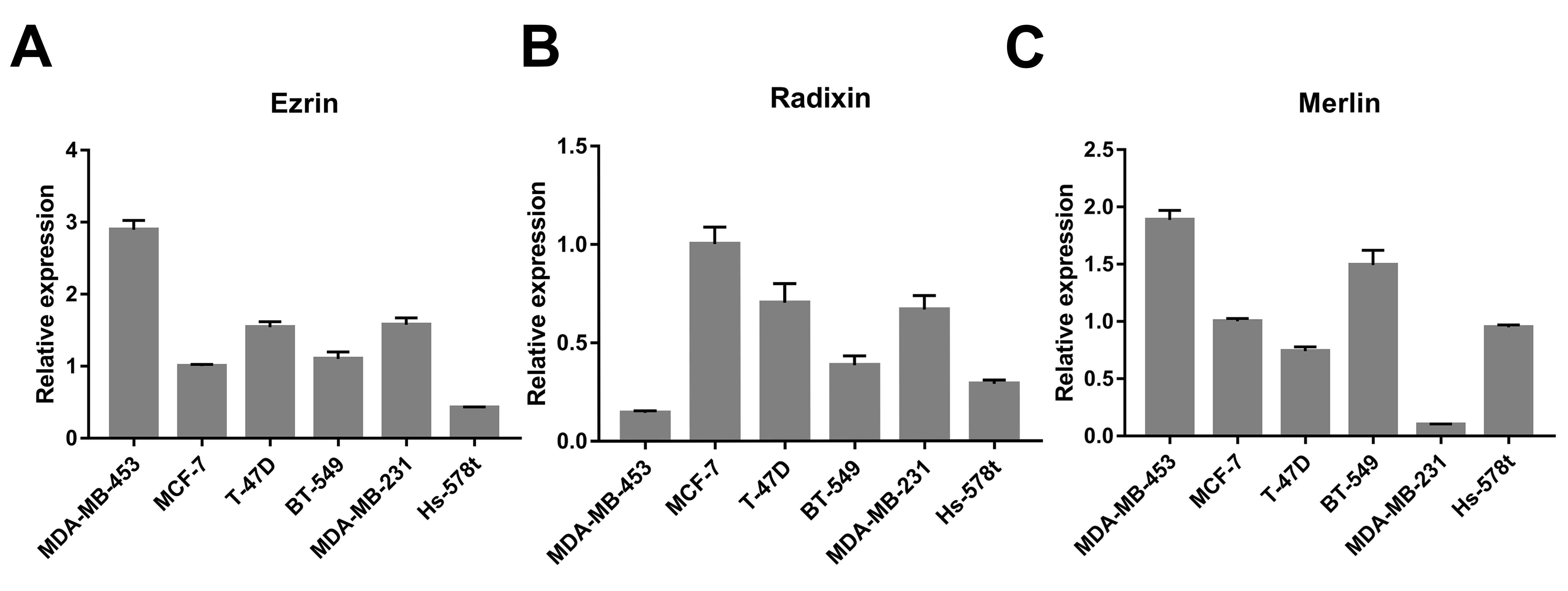

Supplement: Supplementary file 3 — Additional file 3: Figure S2. Expression of Ezrin, Radixin and Merlin in breast cancer cell lines. (A-C) Expression levels of Ezrin, Radixin and Merlin in six breast cancer cell lines. [file 12935_2020_1663_MOESM3_ESM.tif]

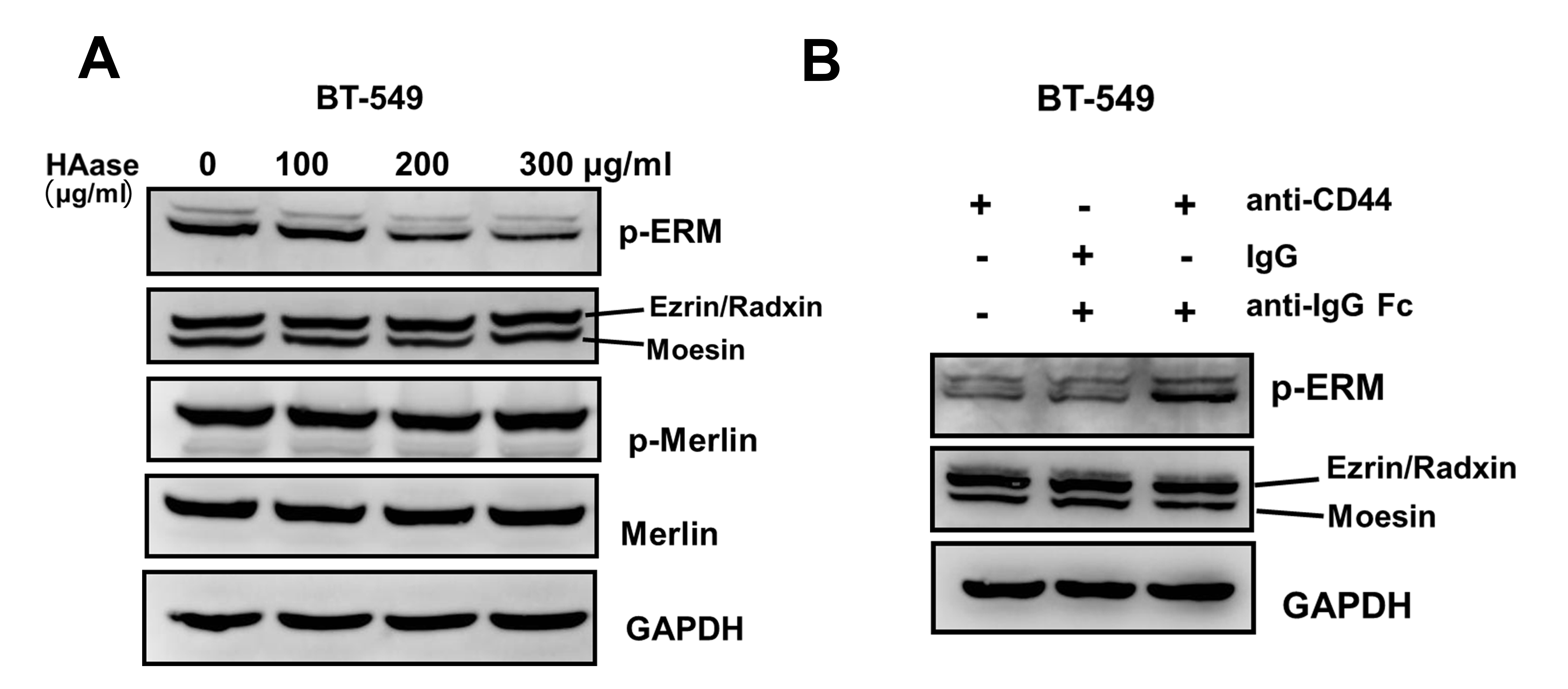

Supplement: Supplementary file 4 — Additional file 4: Figure S3. Alteration of CD44 cross-linking status on the expression level of P-Moesin in BT-549 cells. (A) Effects of hyaluronidase at different concentrations on the expression of ERM and Merlin and their phosphorylated forms in BT-549 cells; (B) p-Moesin and ERM expression after CD44 antibody mediated cross-linking in BT-549 cells. [file 12935_2020_1663_MOESM4_ESM.tif]

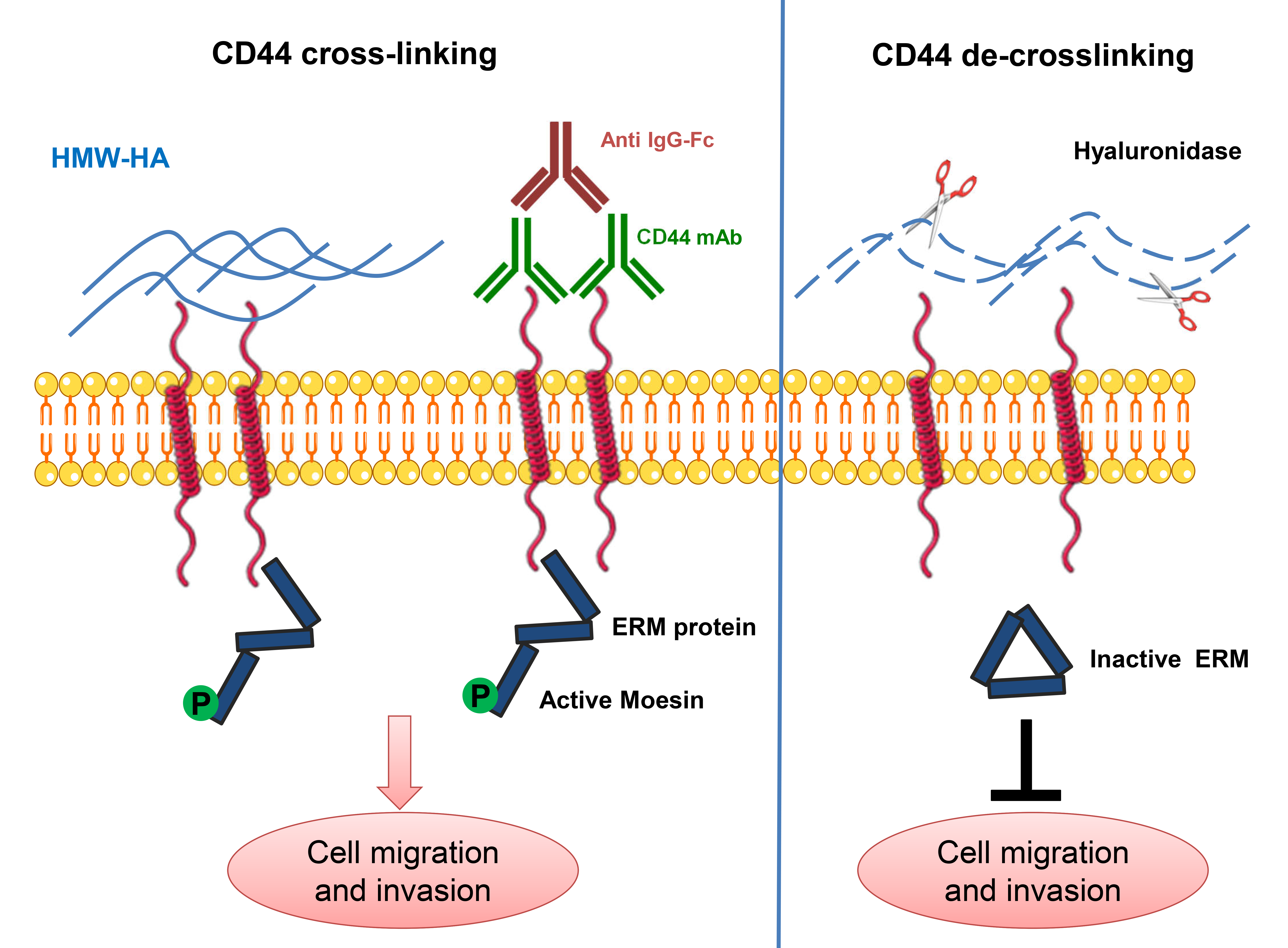

Supplement: Supplementary file 5 — Additional file 5: Figure S5. Working model of the CD44 cross-linking : p-Moesin regulatory axis in breast cancer. [file 12935_2020_1663_MOESM5_ESM.tif]
